# Shared decision making in type 2 diabetes with a support decision tool that takes into account clinical factors, the intensity of treatment and patient preferences: design of a cluster randomised (OPTIMAL) trial

**DOI:** 10.1186/s12875-015-0230-0

**Published:** 2015-02-27

**Authors:** Henk den Ouden, Rimke C Vos, Carla Reidsma, Guy EHM Rutten

**Affiliations:** Julius Centre for Health Sciences and Primary Care, University Medical Centre Utrecht, Str. 6.131, PO Box 85500, 3508 GA Utrecht, The Netherlands

**Keywords:** Decision support tool, Personalised diabetes treatment, Shared decision making, Goal setting

## Abstract

**Background:**

No more than 10-15% of type 2 diabetes mellitus (T2DM) patients achieve all treatment goals regarding glycaemic control, lipids and blood pressure. Shared decision making (SDM) should increase that percentage; however, not all support decision tools are appropriate. Because the ADDITION-Europe study demonstrated two (almost) equally effective treatments but with slightly different intensities, it may be a good starting point to discuss with the patients their diabetes treatment, taking into account both the intensity of treatment, clinical factors and patients’ preferences. We aim to evaluate whether such an approach increases the proportion of patients that achieve all three treatment goals.

**Methods:**

In a cluster-randomised trial including 40 general practices, that participated until 2009 in the ADDITION Study, 150 T2DM patients 60–80 years, known with T2DM for 8-15 years, will be included. Practices are randomised a second time, i.e. intervention practices in the ADDITION study could be control practices in the current study and vice versa. For the GPs from the intervention group a 2-hour training in SDM was developed as well as a decision support tool to be used during the consultation. GPs plan the first visit with the patients to decide on the intensity of the treatment, personalised targets and the priorities of treatment. The control group will continue with the treatment they were allocated to in the ADDITION study. Follow-up: 24 months. The primary outcome is the proportion of patients who achieve all three treatment goals. Secondary outcomes are the proportion of patients who achieve five treatment goals (HbA1c, blood pressure, total cholesterol, body weight, not smoking), evaluation of the SDM process (SDM-Q9 and CPS), satisfaction with the treatment (DTSQ), wellbeing and quality of life (W-BQ12, ADD QoL-19), health status (SF-36, EQ-5D) and coping (DCMQ). The proportions of achieved treatment goals will be compared between both groups. For the secondary outcomes mixed models will be used. The Medical Research Ethics Committee of the University Medical Centre Utrecht has approved the study protocol (Protocol number: 11-153).

**Discussion:**

This trial will provide evidence whether an intervention with a multi-faceted decision support tool increases the proportion of achieved personalised goals in type 2 diabetes patients.

**Trial registration:**

NCT02285881, November 4, 2014

## Background

Successful prevention of complications in the increasing number of patients with type 2 diabetes (T2DM) appears to be difficult. In primary care no more than 10-15% of the patients with T2DM achieve all three treatment targets (glycaemic control, lipids, blood pressure) [1-6]. For separate targets much higher percentages of about 30-70% are reported [2-6]. Therefore, it has been suggested that a more personalised and patient centred approach might increase the proportion of patients who successfully reach all their treatment targets [7,8]. To personalise treatment targets for HbA1c, lipids, blood pressure, weight loss and smoking cessation, several factors have to be considered in order to encourage active participation of patients in evidence based clinical decision making.

Physicians are advised to consider the patients’ age, motivation, risk of hypoglycaemia, diabetes duration, comorbidity and established vascular complications in setting the glucose control target [8,9]. For statin therapy, physicians should consider the patient’s individual cholesterol level, his/her risk for cardiovascular mortality, age and T2DM duration [10]. The blood pressure target for patients with T2DM is below 140/80 [11,12], but for older patients a less strict treatment target may result in a higher survival rate [13]. With regard to weight control, physicians have to consider the side effects of weight gain of medication. Intensive lifestyle modification remains an elusive gold standard for weight reduction [14] Although smoking cessation has been associated with weight gain, it is recommended as a routine component of the treatment of diabetes; however, evidence to guide best practice is limited [15].

To achieve and maintain treatment targets, not only individual clinical characteristics should be considered, but also patients’ preferences for treatment intensity. Generally speaking, the doctor is the expert on medicine, while the patient is the expert on his or her priorities [16]. Shared decision making (SDM) is an approach that takes into account both the clinical evidence for treatment goals as well as the patient’s preferences. SDM is defined as ‘an approach where clinicians and patients make decisions together, using the best available evidence’ [17]. It promotes patient autonomy and patient engagement in the treatment decision making by giving the patient an active role in weighting the benefits and harms of more than one evidence based treatment option [17]. Although SDM is promising for patients with chronic diseases by setting realistic treatment targets, such an extensive approach had not been broadly studied in T2DM patients before 2008 [18]. Recently the effects a patient oriented decision aid for SDM and goal setting in T2DM patients on patient empowerment and treatment decisions have been published [19]. No effect was found on empowerment, the decision aid was not used to measure the effect on clinical outcomes and achievement of goals.

Because SDM is especially useful when there are two or more equally beneficial treatment options, the results of the ADDITION-Europe study, in which the Netherlands participated, could be used in a SDM approach in patients with T2DM. The ADDITION study included screen detected T2DM patients and compared an intensive multifactorial treatment of HbA1c, cholesterol, blood pressure and body weight with less intensive usual care according to national guidelines. The intensive treatment was associated with a significant increase in prescribed medications and a non-significant 17% reduction of cardiovascular events and death after 5 years. However, the rate of cardiovascular events seemed to diverge after 4 years of follow-up. It was concluded that intensified treatment and treatment according to national guidelines can theoretically be equally effective [1]. Based on the results of the ADDITION study, it is questionable which evidence based treatment advice the diabetes care provider should give to the T2DM patient. There is no decisive evidence for either option. This situation is very appropriate for treatment decisions that incorporate the patient’s preferences.

In a recent statement of both the American Diabetes Association (ADA) and the European Association for the Study of Diabetes (EASD) personalised and patient-centred care is mentioned as the cornerstone of the treatment of patients with T2DM. The use of a decision support tool is strongly advocated [8]. A decision support tool can encourage active patient participation in many evidence-based healthcare decisions [20-22]. In the last decade decision support tools have been developed to support the achievement of cardiometabolic goals and to select patient-centred treatment options for lifestyle modifications or medication use [8,23-28]. However, most of them focus on a single risk factor [25], on only the patients’ preferences [26,27] or on some individual clinical characteristics [28]. We hypothesise that a decision support tool that takes into account both treatment intensity, patient’s clinical characteristics and patient’s preferences can facilitate a SDM process and will be effective in achieving treatment targets. We aim to evaluate whether such an approach increases the proportion of treatment targets that T2DM patients achieve.

The following research questions are addressed:

1. What is the effect of shared decision making with a multi-faceted decision support tool on the percentage of patients with T2DM that achieve all individualised treatment targets for HbA1c, blood pressure and LDL-cholesterol; and to identify determinants for achieving all individualised treatment targets.
2. What is the effect of shared decision making with a multi-faceted decision support tool on treatment satisfaction, quality of life, health status, well-being and on coping styles?
3. What is the level of SDM-knowledge/attitude of the GPs from both treatment groups after 24 months?

## Methods

### Study design and setting

**Figure 1:**
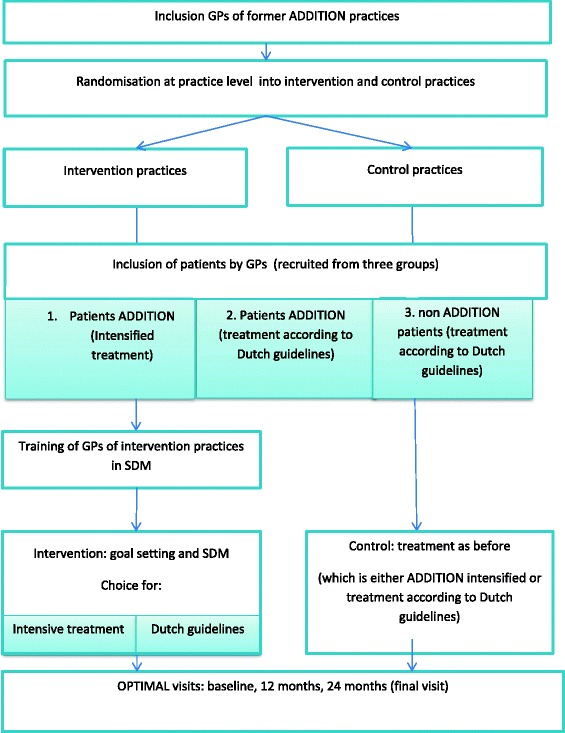
**Flow chart.**

The OPTIMAL study is a cluster-randomised trial with randomisation at practice-level and two years follow-up. We developed an intervention to promote SDM with a decision support tool based on the results of the above mentioned ADDITION-Europe study. Since for an optimal SDM approach physicians should have some experience with all treatment options, patients are recruited from the 79 general practices that participated in the ADDITION study [29]. For the OPTIMAL study practices are randomised again (Figure 1). GPs in the intervention group were trained in SDM (see further).

### Practices and patients

Eligible GPs are those who included at least included one patient in the ADDITION-study. For the OPTIMAL study each GP should include at least two more or less comparable patients: 1. Former ‘ADDITION’ patients diagnosed with T2DM in 2002-2004 by screening, aged between 50-70 years at that time and having participated in the ADDITION study that ended in 2009; 2. Patients between 60 and 80 years in 2012-2014, known with type 2 diabetes for 8-12 years but not diagnosed in the ADDITION study. Patients will be excluded if they have a history of alcoholism, drug abuse, psychosis, personality disorder or another emotional, psychological or intellectual problem that is likely to invalidate informed consent, or limit the ability of the individual to comply with the protocol requirements. Also, patients with a limited life expectancy will not be approached.

### Randomisation

Randomisation is executed at the research center at practice-level, without any stratification. It is not possible to blind participants and GPs for the treatment allocation. Practices are randomised a second time, i.e. intervention practices in the ADDITION study could be control practices in the current study and vice versa.

### Intervention

1. The decision support tool.
  The OPTIMAL decision support tool is a simple paper-based tool, aimed to be easy to use for both the GP and the patients. It should be used to discuss the treatment options and the prioritising of treatment targets during the first visit and the 12- and 24-months follow-up visits. The tool consists of three steps: 1) considering the pros and cons of two almost equally effective evidence based multifactorial treatments, namely the intensified ADDITION protocol and the protocol derived from the 2006 Dutch guidelines for GPs [1] (for details see Figure 2, step 1b), and the shared decision on which option will be chosen; 2) prioritising of treatment targets according to the chosen option, and 3) treatment selection (medication or lifestyle) to achieve the treatment targets. For detailed information about the two treatment options and the different targets see Figure 2.

**Figure 2:**
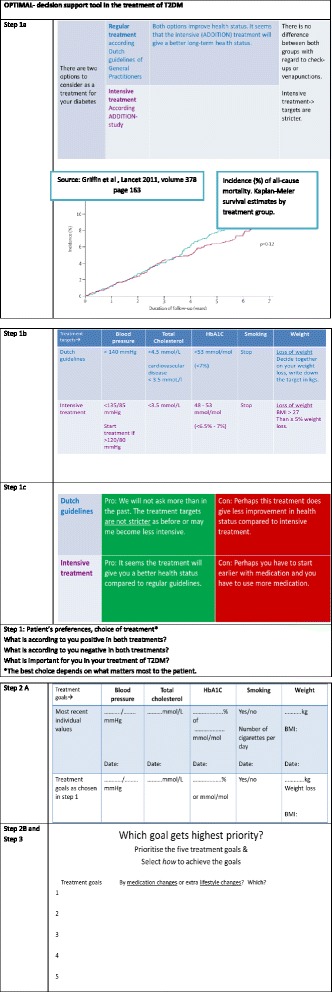
**Decision support tool.**
2. Visits during the OPTIMAL study.
  During the first visit, and guided by the decision tool, the GP and the patient discuss the two evidence based T2DM treatments. The GP will explain the pros and cons of each option, and the treatment targets that should be achieved, depending on the option that is chosen. The GP should explain that both treatments are equally suitable for treating T2DM. Next, the patient’s preferences and lifestyle habits are discussed against the background of the patient’s most recent values of HbA1c, lipids, blood pressure, body weight and smoking habits. Then the GP and the patient will together decide on the preferred treatment option and the accompanying treatment targets. For a patient who underwent the intensified treatment in the ADDITION study and did not change it after the end of ADDITION, this visit provides him/her the opportunity to choose a less intensive treatment. Also the other way around is possible. The patient will prioritise the five treatment targets (HbA1c, cholesterol, blood pressure, body weight, smoking habits) for the first 12 months. The target with the highest priority will get the most attention and require the most effort of both the patient and GP. There will be no pre-defined way in how the patients should reach their targets; the patient and the GP need to determine this way together in a final step in the decision process. The patient and GP decide whether medication changes and/or lifestyle changes should be made in order to reach the prioritised targets. During the second and third OPTIMAL visit (12 and 24 months later) the patient and GP evaluate the decisions they made during the first visit using the decision support tool. The priority of the treatment targets can be changed during these visits, but not the choice between the two treatment options (ADDITION protocol or the less intensive protocol). During the third and final visit the patient and the GP will decide whether or not to keep to the chosen treatment option. Between the first and third visit, three monthly T2DM visits will take place either with the GP or the practice nurse.

### Training

The GPs from the practices randomised to the SDM-treatment arm are trained in the SDM approach during a two hours training session. During this session the study protocol is discussed as well as the SDM principle (see further) and the OPTIMAL decision support tool. By use of role-plays the SDM process will be practiced by the GPs.

### Control group

Patients in the control practices will receive treatment-as-before. Also, the GPs will not be asked to engage in a SDM process, nor be trained to do this and they will not be offered the decision support tool. The GP will treat the patients as they were used to since the ending of the ADDITION study (2009), either following the national guidelines or the ADDITION intensive treatment algorithm, each with their respective targets [1].

### Outcome measures

Primary outcome will be the proportion of patients that achieve all the three treatment goals for HbA1c, blood pressure, and total cholesterol .In addition to identify determinants of better performing patients for the primary outcome will be performed taken into account the interaction of SDM with age, gender, education level, duration of diabetes and comorbidities.

Secondary outcomes:

- The proportion of patients that achieve the all five treatment goals for HbA1c, blood pressure, total and LDL- cholesterol, body weight, and smoking.
- The following patient reported outcomes: diabetes treatment satisfaction, perceived quality of life, health status, well-being, and coping style.
- A process evaluation of the shared decision making ability of the general practitioners during the complete study. Outcome on GP level: the level of SDM-knowledge/attitude.

### Measurements, data collection

Data about the patient’s socio-demographic background, the level of education, smoking status and whether the patient lives alone or together will be collected by patients’ self-report at baseline. HbA1c, blood pressure, body weight, total cholesterol and LDL-cholesterol, and smoking habits as well as patient characteristics (age, gender, duration of diabetes and comorbidities) will be reported every year by the GPs on a specific Case Report Form in both groups.

Blood pressure is measured by two measurements after at least 10 minutes rest, while participants are seated with the cuff on the predominant arm at the level of the heart, using an automatic sphygmomanometer. Height and body weight are measured in light indoor clothing and without shoes using a fixed rigid stadiometer and a scale respectively. Laboratory results (HbA1c, LDL-cholesterol) were obtained with case report from the GPs electronic records.

Participants in both groups will be asked to complete and return the following questionnaires baseline and after 24 months at home.

- the Diabetes Treatment Satisfaction Questionnaire (DTSQ) [30] which includes 8 items; scores range from 0 (very dissatisfied) to 6 (very satisfied), totally from 0 to 48. The DTSQ is reliable, valid and sensitive to change in diabetes patients [30];
- the Audit of Diabetes Dependent Quality of Life (ADD QoL-19), which measures the perceived impact of diabetes on the quality of life; it includes 19 items, ranging from -3 to 3 on different questions, with 0 as the neutral score. Scores below 0 reflect a negative influence of the item on quality of life, and all above 0 reflect positive influences. The impact scores are weighted (impact rating x importance rating), so the actual scores per item can range from -9 to 9. The ADDQoL-19 has good psychometric properties and provides clinicians and researchers with a useful tool for comprehensively assessing quality of life in adults with T2DM [31];
- the Well-Being Questionnaire (W-BQ12) that consists of 12-items in three 4-item subscales: negative well-being (item 1-4, higher score reflects a greater sense of negative well-being), energy (items 6 and 7 are reversed, and then together with 5 and 8 form the total amount of energy) and positive well-being (items 9-12, the higher the score the greater the sense of positive well-being). The total score ranges from 0 to 36 and is called the general well-being score. Higher scores indicate a higher overall sense of well-being [32];
- the European Quality of Life (EQ-5D) questionnaire, that covers 5 dimensions (mobility, self-care, usual activities, pain/discomfort and anxiety/depression) and a Visual Analogue Scale (VAS) where respondents can rate their health. Item scores range from 1-3, and a 5-digit health profile is formed, placing the 5 numbers behind each other. It is a well-validated, reliable and responsive instrument for health measurement in patients with a wide range of medical conditions. Values found in the UK have been validated for the Netherlands [33-35];
- the Short Form-36, a validated 36-item instrument for the self-evaluation of health status with eight subscales: Physical Functioning (10 items), Role Physical (4 items), Bodily Pain (2 items), General Health (5 items), Vitality (4 items), Social Functioning (2 items), Role-Emotional (3 items) and Mental Health (5 items). These scales can be summarised in Physical Health and Mental Health. The 36 items differ in the scoring ranges. The Dutch version has proved to be a practical, reliable and valid instrument [36,37];
- the Diabetes Coping Measurement Questionnaire (DCMQ), consisting of 21 items in 4 subscales: spirit coping, avoidance coping, passive resignation coping and diabetes integration coping. Overall scores range from 7 to 35. The items are measures on a 5-point Likert scale, ranging from 1 (“disagree”) to 5 (“agree strongly”) or the other way around from 1 (‘I strongly agree’) to 5 (I ‘disagree’). Higher scores on tackling spirit and diabetes integration indicate more adaptive coping. Higher scores on passive resignation and avoidance indicate poor coping [38].

A process evaluation of the shared decision making ability of the general practitioners will be measured in the intervention group at baseline and after 12 months, and in both treatment groups at 24 months. Both the patients and GPs will be asked to complete The Shared Decision Making Questionnaire, both the patient (SDM-Q9-patient) and GP (SDM-Q9-doc) version will be used to evaluate this process.

The Shared Decision Making Questionnaire (both SDM-Q9 versions) [39-41] includes 9 items with ratings from 0 (completely disagree) to 6 (completely agree); the total score ranges from 0 to 54. It is a continuous scale, and the questionnaire developers did not describe any thresholds for ‘bad’ or ‘good’ SDM. Over the last years the SDM-Q9 has become a frequently used instrument in clinical practice. It has been translated into several languages. Internal consistency has been assessed for the Spanish and Dutch version (Rodenburg-Vandenbussche S, Pieterse AH, Kroonenberg PM, Stiggelbout AM,et al.: Further validation of the SDM-Q-9 and SDM-Q-Doc: psychometric properties in Dutch patients from primary and secondary care. Submitted). Item discrimination parameters were above 0.4 for all but one item. An analysis of internal consistency yielded a Cronbach’s α of 0.88 [39]. The Dutch version is currently being validated [41]. A modified version of the Control Preferences Scale (CPS) is used to determine the experienced role of decision making of the GP and patient. The CPS measures at a 5-point Likert scale, and has shown good reliability and validity [41,42]. The original Control Preference Scale by Degner [41], was developed to measure preference for involvement and is one of the most commonly used instruments to assess preferred decisional role [41,42].

Subsequently, the GPs of both groups (the intervention group trained, the control group not) will audio- or videotape one of their yearly consultations with a T2DM patient. They can choose the consultation to be taped themselves. These tapes will be evaluated by two independent observers, making use of the SDM-Q9, to assess the extent to which the GP is likely to involve his/her patients in the diabetes treatment.

### Sample size

As stated in the Introduction, only 10-15% of T2DM patients achieve all three treatment targets for both HbA1c, lipids and blood pressure. We estimate the percentage of patients that already has reached three targets at the start of the study will be approximately 10%. We hypothesise that in the OPTIMAL study, after two years of follow-up, the intervention group will show an increase of this percentage until 30% (about 10% increase per year), whereas in the treatment-as-before-group this percentage will stay at 10%. Assuming a two-sided significance level of 5%, with alpha 0.05 and power of 80% and with a drop-out of 10%, 65 patients will be needed in each treatment group (Department of Statistics Sample Size Calculator, University of British Columbia). Because the OPTIMAL study is a cluster randomised study the sample size will require an correction for the cluster effect. The used correction factor is equal to [1 + (m - 1) r], where ‘m’ is the total amount of eligible patient per practice (approximately 6), and ‘r’ the within-cluster correlation coefficient. For ‘r’ we use a within-cluster correlation coefficient of 0.025, based on the cluster correlation found in the ADDITION-Europe study. When taken the cluster effect (1.125) in to account, 73 patients per group are needed.

### Statistical analyses

Intention-to-treat analyses (ITT) will be performed to examine between-group differences. Generalized linear models will be used to correct for clustering at practice level. The proportions of achieved treatment goals of HbA1c, blood pressure, LDL-cholesterol within each study group will be estimated by calculating relative risk. The same applies to the difference between groups in the proportion of patients which achieved all the five above mentioned treatment goals. A p-value of <0.05 is considered statistically significant. To identify patients who show better results after the SDM process, the analysis for the primary outcome will be repeated with taken into account interaction of SDM with age, gender, education level, duration of diabetes and comorbidities. Within and between group differences in treatment satisfaction, perceived quality of life, health status, well-being, and coping style between baseline and 24 months follow-up will be analysed by using paired t-tests and mixed models respectively. We will add random effects for patient and practice.

The SDM-Q9 will be analysed in the intervention group by using paired t-tests. Mixed models will be used to study the between groups differences after 24 months. We will add random effects for patient and practice.

To evaluate the SDM proces, the tapes will be evaluated by two independent observers by making use of the SDM-Q9.

The Medical Research Ethics Committee of the University Medical Centre Utrecht has approved the study protocol (Protocol number: 11-153).

## Discussion

The treatment of T2DM is mostly target driven. However, only a low percentage of all patients with T2DM achieve all goals. SDM and goal setting can be useful to increase the percentage of patients that achieve all targets. However, the decision support tool to be used in SDM should likely not only focus on the clinical factors of the patients but also on the patient’s preferences, because each of these variables may affect the optimal treatment targets. Besides, the decision support tool should be used in a SDM process during more than one consultation [8]. The results of the ADDITION trial, in which half of the participants of the current study also participated, offers a unique opportunity to discuss with the patient two almost equally effective treatment strategies.

In the current cluster-randomised controlled trial we will evaluate the effectiveness of such a repeated use of a decision support tool, taking into account both the intensity of treatment, individual clinical factors and the patients’ experiences and preferences. We hypothesise that the SDM process with such a well-balanced decision support tool will improve the percentage of patients that achieve all three individual goals compared to the control group, making SDM really beneficial.

## Abbreviations

SDM: Shared decision making
GP: General practitioner
T2DM: Type 2 diabetes

## References

[1] Griffin SJ, Borch-Johnsen K, Davies MJ, Khunti K, Rutten GE, Sandbaek A (2011). Effect of early intensive multifactorial therapy on 5-year cardiovascular outcomes in individuals with type 2 diabetes detected by screening (ADDITION-Europe): a cluster-randomised trial. Lancet.

[2] Berkowitz SA, Meigs JB, Wexler DJ (2013). Age at type 2 diabetes onset and glycaemic control: results from the National Health and Nutrition Examination Survey (NHANES) 2005-2010. Diabetologia.

[3] Camara S, Bouenizabila E, Hermans MP, Ahn SA, Rousseau MF (2014). Novel determinants preventing achievement of major cardiovascular targets in type 2 diabetes. Diabetes Metab Syndr.

[4] Gaede P, Vedel P, Larsen N, Jensen GV, Parving HH, Pedersen ON (2003). Multifactorial intervention and cardiovascular disease in patients with type 2 diabetes. Engl J Med.

[5] Cleveringa FG, Gorter KJ, van den Donk M, Rutten GE (2008). Combined task delegation, computerized decision support, and feedback improve cardiovascular risk for type 2 diabetic patients: a cluster randomized trial in primary care. Diabetes Care.

[6] Van Hateren KJ, Drion I, Kleefstra N, Groenier KH, Houweling ST, van der Meer K, Bilo HJ. A prospective observational study of quality of diabetes care in a shared care setting: trends and age differences (ZODIAC-19). BMJ Open. 2012;2 10.1136/bmjopen-2012-001387 PMC3432849 22936821

[7] White RD (2012). Patient empowerment and optimal glycemic control. Curr Med Res Opin.

[8] Inzucchi SE, Bergenstal RM, Buse JB, Diamant M, Ferrannini E, Matthews DR, et al. Management of hyperglycemia in type 2 diabetes: a patient-centered approach: position statement of the American Diabetes Association (ADA) and the European Association for the study of Diabetes (EASD). Diabetes Care. 2012;35:1364–79. 10.2337/dc12-0413 PMC3357214 22517736

[9] Ismail-Beigi F, Moghissi E, Tiktin M, Hirsch IB, Inzucchi SE, Genuth S (2011). Individualizing Glycemic Targets in Type 2 Diabetes Mellitus: Implications of Recent Clinical Trials. Ann Intern Med.

[10] Robinson JG (2014). ACC/AHA Cholesterol Guideline for Reducing Cardiovascular Risk: What is so Controversial?. Curr Atheroscler Rep.

[11] Esposito K, Maiorino MI, Bellastella G, Giugliano D (2014). New guidelines for metabolic targets in diabetes: clinician’s opinion does matter. Endocrine.

[12] Standards of Medical Care in Diabetes 2014. Diabetes Care 2014 vol. 37 no. Supplement 1 S14-S80 10.2337/dc14-S014 24357209

[13] De Ruijter W, Westendorp RG, Assendelft WJ, Den Elzen WP, De Craen AJ, Le Cessie S (2009). Use of Framingham risk score and new biomarkers to predict cardiovascular mortality in older people: population based observational cohort study. BMJ.

[14] Niswender K (2010). Diabetes and obesity: therapeutic targeting and risk reduction - a complex interplay. Diabetes Obes Metab.

[15] Nagrebetsky A, Brettell R, Roberts N, Farmer A (2014). Smoking cessation in adults with diabetes: a systematic review and meta-analysis of data from randomised controlled trials. BMJ Open.

[16] Mulley AG, Trimble C, Elwyn G (2012). Stop the silent misdiagnosis: patients' preferences matter. BMJ.

[17] Stiggelbout AM, Van der Weijden T, De Wit MPT, Frosch D, Légaré F, Montori VM (2012). Shared decision making: really putting patients at the centre of healthcare. BMJ.

[18] Joosten EA, Fuentes-Merillas L, de Weert GH, Sensky T, van der Staak CP, de Jong CA (2008). Systematic review of the effects of shared decision-making on patient satisfaction, treatment adherence and health status. Psychother Psychosom.

[19] Denig P, Schuling J, Haaijer-Ruskamp F, Voorham J (2014). Effects of a patient oriented decision aid for prioritising treatment goals in diabetes: pragmatic randomized controlled trial. BMJ.

[20] Barry MJ, Edgman-Levitan S (2012). Shared decision making—the pinnacle of patient-centred care. N Engl J Med.

[21] Coulter A, Collins A. Making shared decision-making a reality: no decision about me, without me. 2011. www.kingsfund.org.uk/publications/nhs_decisionmaking.html.

[22] International Patient Decision Aids Standards Collaboration. Criteria for judging the quality of patient decision aids. 2005. www.ipdas.ohri.ca/IPDAS_checklist.pdf.

[23] Wilkinson MJ, Nathan AG, Huang ES (2012). Personalized Decision Support in Type 2 Diabetes Mellitus: Current Evidence and Future Directions. Curr Diab Rep.

[24] Rodbard D, Vigersky RA (2011). Design of a decision support system to help clinicians manage glycemia in patients with type 2 diabetes mellitus. J Diabetes Sci Technol.

[25] Mann DM, Ponieman D, Montori VM, Arciniega J, McGinn T. The statin choice decision aid in primary care: a randomized trial. Patients Educ Couns. 2010;80:138–40. 10.1016/j.pec.2009.10.008 19959322

[26] Mullan RJ, Montori VM, Shah ND, Christianson TJ, Bryant SC, Guyatt GH (2009). The diabetes mellitus medication choice decision aid: a randomized trial. Arch Intern Med.

[27] Corser W, Holmes-Rovner M, Lein C, Gossain V (2007). A shared decision-making primary care intervention for type 2 diabetes. Diabetes Educ.

[28] Holbrook A, Thabane L, Keshavjee K, Dolovich L, Bernstein B, Chan D, et al. Individualized electronic decision support and reminders to improve diabetes care in the community COMPETE II randomized trial. CMAJ. 2009;181:37–44. 10.1503/cmaj.081272 PMC2704409 19581618

[29] Janssen PGH, Gorter KJ, Stolk RP, Rutten GEHM (2009). Randomised controlled trial of intensive multifactorial treatment for cardiovascular risk in patients with screen-detected type 2 diabetes: 1-year data from the ADDITION Netherlands study. Br J Gen Pract.

[30] Bradley C (1994). Diabetes Treatment Satisfaction Questionnaire (DTSQ). Handbook of Psychology and Diabetes: A Guide to Psychological Measurement in Diabetes Research and Practice.

[31] Bradley C, Todd C, Gorton T, Symonds E, Martin A, Plowright R (1999). The development of an individualized questionnaire measure of perceived impact of diabetes on quality of life: the ADDQoL. Qual Life Res.

[32] Pouwer F, van der Ploeg HM, Ader HJ, Heine RJ, Snoek FJ (1999). The 12-item well-being questionnaire. An evaluation of its validity and reliability in Dutch people with diabetes. Diabetes Care.

[33] Janssen MF, Lubetkin EI, Sekhobo JP, Pickard AS (2011). The use of the EQ-5D preference-based health status measure in adults with Type 2 diabetes mellitus. Diabet Med.

[34] The EuroQoL Group (1990). EuroQol-a new facility for the measurement of health-related quality of life. Health Policy.

[35] Lamers LM, McDonnell J, Stalmeier PF, Krabbe PF, Busschbach JJ (2006). The Dutch tariff: results and arguments for an effective design for national EQ-5D valuation studies. Health Econ.

[36] Aaronson NK, Muller M, Cohen PD, Essink-Bot ML, Fekkes M, Sanderman R (1998). Translation, validation, and norming of the Dutch language version of the SF-36 Health Survey in community and chronic disease populations. J Clin Epidemiol.

[37] Ware JEJr&SCD (1992). The MOS 36-item short-form health survey (SF-36). I. Conceptual framework and item selection. Med Care.

[38] Welch G, Bradley C (1994). The Diabetes Coping Measure: A measure of cognitive and behavioural coping specific to diabetes. Handbook psychology and diabetes: A guide to psychological measurement in diabetes research and practice.

[39] Kriston L, Scholl I, Holzel L, Simon D, Loh A, Harter M (2010). The 9-item Shared Decision Making Questionnaire (SDM-Q-9) Development and psychometric properties in a primary care sample.. Patient Educ Couns.

[40] Scholl I, Kriston L, Dirmaier J, Buchholz A, Härter M (2012). Development and psychometric properties of the Shared Decision Making Questionnaire–physician version (SDM-Q-Doc). Patient Educ Couns.

[41] Degner LF, Sloan JA, Venkatesh P (1997). The Control Preference Scale. Can J Nurs Res.

[42] Kasper J, Heesen C, Köpke S, Fulcher G, Geiger F (2011). Patients’ and observers’ perceptions of involvement differ. Validation study on inter-relating measures for shared decision making. PLoS One.

